# Holographic Whole‐Object Photopolymerization Preserving Director Alignment in Liquid Crystalline Actuators

**DOI:** 10.1002/adma.202519970

**Published:** 2026-01-29

**Authors:** Lovish Gulati, Junhee Lee, Reza Norouzikudiani, Jianfeng Li, Carlos Sánchez Somolinos, Antonio DeSimone, Kai Melde, Alexander Song, Peer Fischer

**Affiliations:** ^1^ Max Planck Institute for Medical Research Heidelberg Germany; ^2^ Institute for Molecular Systems Engineering and Advanced Materials Heidelberg Germany; ^3^ The BioRobotics Institute Scuola Superiore Sant'Anna Pontedera Italy; ^4^ SISSA – Scuola Internazionale Superiore di Studi Avanzati Trieste Italy; ^5^ Instituto de Nanociencia y Materiales de Aragón (INMA) CSIC‐Universidad de Zaragoza Departamento de Física de la Materia Condensada Zaragoza Spain; ^6^ Centro de Investigación Biomédica en Red de Bioingenieria Biomateriales y Nanomedicina (CIBER‐BBN) Madrid Spain; ^7^ Center for Nanomedicine Institute for Basic Science (IBS) Seoul Republic of Korea; ^8^ Department of Nano Biomedical Engineering (NanoBME) Advanced Science Institute Yonsei University Seoul Republic of Korea

**Keywords:** 3D printing, director alignment, holographic microlithography, liquid crystalline structures, single brief exposure

## Abstract

Liquid crystalline (LC) crosslinked polymeric structures are promising for soft robotic applications, as their actuation profile is intrinsically encoded and results from the structure's shape and the LC molecular orientation (director). However, it remains challenging to fabricate 3D objects and at the same time control the director orientation within the 3D structure. Liquid crystalline molecules are commonly aligned using modified surfaces or electric/magnetic fields. However, additive manufacturing methods may locally distort the director, when fabricating 3D objects. Here, holographic microlithography is employed to form entire connected 3D objects in a single exposure by cross‐linking the LC, while allowing the director orientation within the object to be freely controlled. This enables us to independently choose a global director orientation and then realize complex 3D geometries in a single fabrication step. This approach avoids the director distortion present in sequential 3D printing and lithography methods, and allows for complex actuation profiles that are directly linked to the 3D director orientation. The method presented herein permits the rapid fabrication of complex 3D connected LC structures while preserving the molecular order, and thereby enables the fabrication of more complex 3D soft actuators.

## Graphical abstract

Holographic microlithography enables the fabrication of fully connected 3D liquid crystalline structures in a single exposure. The process maintains precise control over the director orientation within the entire object, preventing local director distortions typically observed in sequential printing. The resulting structures exhibit complex actuation profiles directly linked to their 3D director configuration.

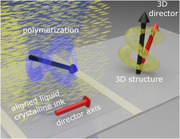

## 1 Introduction

Liquid crystalline (LC) crosslinked polymeric structures form a class of soft robotic actuators that show mechanical deformations under external stimuli based on their geometry and programmed director profile [1, 2, 3, 4, 5, 6, 7, 8, 9, 10, 11]. Complex 3D actuation requires techniques that can form user‐defined 3D shapes with high precision, while preserving molecular order and allowing control over the director orientation within the LC actuators [12, 13, 14, 15, 16]. However, it is still challenging to print structures that have a complex geometry and at the same time possess a defined 3D director profile. Commonly used methods to fabricate macro/micro actuators are direct ink writing (DIW) [13, 14, 15, 17, 18, 19, 20, 21], two‐photon laser printing (2PLP) [16, 22, 23, 24, 25, 26], and digital light processing (DLP) [7, 27, 28, 29]. Even though these techniques are very well established, the additional demands posed by having to control the molecular order is challenging and limits the range of LC actuators that can be fabricated. For instance, in DIW, the ink is extruded by a nozzle such that the mesogens shear‐align during extrusion, but this necessarily causes the director to always follow the direction of flow. The shape is then restricted by writing the ink along optimized, shape‐filling paths to fabricate grippers, swimmers, folding structures or adaptive optical elements [13, 14, 15, 17, 18, 19]. Furthermore, significant director distortions can occur at the interfaces between adjacent printed paths [13, 14, 17, 20, 21, 30]. While isolated fibers would ideally exhibit a uniform director aligned along their symmetry axis, the interfacial regions between neighboring filaments often show alignment distortions. These arise from shear effects during the consecutive deposition of adjacent fibers, which perturb the otherwise uniform alignment and lead to local mismatches in director orientation. The attainable resolution in extrusion printing is typically limited by the nozzle diameter, making it challenging to fabricate fine features or small geometries that nevertheless show good alignment. Melt electrowriting has been shown to achieve much finer filaments, however, it is limited to 2D patterns, and the stacked filaments lead to severe distortions in the vertical direction while also requiring well‐controlled physicochemical properties and writing parameters [31]. Furthermore, DIW requires specific viscoelastic properties of the ink, making some inks with high viscosity challenging to extrude through nozzles with small openings.

A second technique to create 4D LC structures with complex shapes and very fine resolution is 2PLP, a serial printing method requiring special inks [16, 22, 23, 24, 25, 26]. Using quasi‐static electric fields or printing inside barriers can be used to force the director alignment during serial 2PLP printing [16, 22, 23]. Digital photopolymerization techniques, particularly two‐photon polymerization direct laser writing, have been widely used to fabricate 1D, 2D, and 3D liquid crystalline elastomer microstructures with high spatial resolution and precise structural control [32, 33]. These approaches rely on sequential continuous writing processes, in contrast to the single exposure volumetric fabrication strategies demonstrated in the present work.

There are also other methods to prepare LC actuators with complex director patterns, such as hybrid surface alignment [34, 35], magnetic‐field‐assisted alignment [36], photoalignment with polarized light [37, 38, 39], alignment induced by curved or patterned surfaces [35], and droplet confinement [40]. Similarly, magnetic‐field alignment of LC monomers and microfluidic fabrication strategies have also been employed to realize LCE actuators with complex director profiles and shape transformations [41, 42]. While these approaches enable rich director profiles, they generally do not provide independent control over both the global 3D director field and the free‐form 3D shape of the actuator within the same fabrication process.

DLP enables fast layer‐by‐layer fabrication of LC structures and director alignment using high magnetic field strengths [27, 28, 29]. However this approach required additional temperature control. In general, sequential printing methods accompany director distortions at the interface between printed regions, leading to boundary effects and a potentially compromised uniformity in the director profile [7], which has also been observed in a number of other printing methods [13, 17, 20, 21]. Across all traditional alignment and printing techniques, it remains challenging to print complex 3D LC microstructures at room temperature while freely setting the director without creating distorted director interfaces.

The successful adaptation of light‐based additive manufacturing methods to 3D LC actuator fabrication envisions further incorporation with advanced techniques, such as volumetric additive manufacturing [43, 44]. Holographic lithography shapes intensity patterns to fabricate 2D and 3D structures by employing phase masks and by controlling interference patterns [45, 46, 47, 48, 49], which can be optimized using computer generated holography (CGH) [50, 51, 52]. Recent advances in CGH algorithms have enhanced the fidelity of 3D holograms compared to earlier algorithms, and they also have shown a marked reduction in speckle noise, which envisions their application in light‐based fabrications.

Here, we propose a new fabrication technique for 3D LC micro‐structures without distorting LC molecular orientations during the printing. We modulate the phase of a coherent laser to project a 3D intensity pattern (3D image) into the LC ink. The 3D light pattern polymerizes the object as a single unit with a single brief exposure, thereby it avoids the generation of interfaces within the structure. The global director orientation is defined by the rubbed alignment substrates. However, the relative orientation of the holographically projected 3D pattern can be chosen independently of the LC ink alignment, enabling identical 3D structures to be fabricated using inks with different global director orientations and, consequently, distinct actuation profiles. We utilize Non‐Convex Optimization for VOlumetric CGH (NOVO‐CGH) algorithm [53] to generate a 3D volumetric light pattern with high contrast between bright regions and the dark background. The photopolymerization proceeds only efficiently in those bright regions where they received photo‐energy higher than a photopolymerization threshold. The intensity distribution of the 3D image allows the UV‐curing to occur almost simultaneously across the entire LC structure. As a result, it resolves the director distortion problem that commonly occur in serial printing methods.

## 2 Results

### 2.1 Holographic Microlithography

**FIGURE 1:**
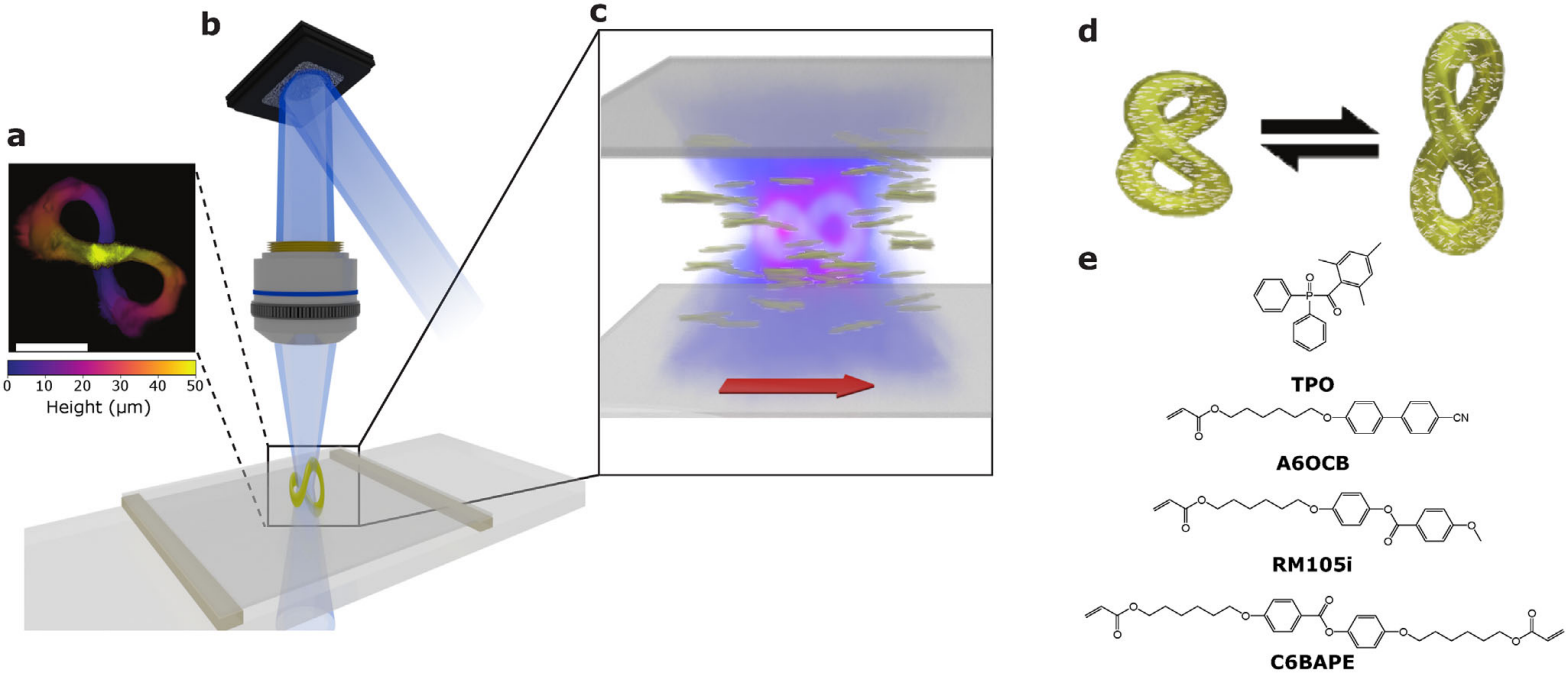
Concept for liquid crystal (LC) structures fabrication via holographic microlithography. (a) Experimentally measured 3D light pattern in the shape of a infinity‐loop generated by (b) the holographic microlithography set up presenting only key components: a spatial light modulator (SLM), an objective lens, and a laser. (c) A 3D liquid crystal microstructure is printed in the regions, where the light intensity exceeds a threshold to initiate photo‐polymerization (regions with magenta color). A rubbed surface (direction ‐ red arrow) induces LC molecular alignment (nematic phase). (d) One step photo‐polymerization fixes the aligned nematic LC within the printed structures, as indicated white directors inside the yellow printed structure. The printed structure deforms when its molecular alignment is disturbed as a response to external stimuli. (e) Chemicals used in the LC ink formulation for this experiment (for details, see Experimental Section).

Holographic microlithography fabricates 3D LC structures by briefly exposing a 3D light field to a photo‐curable LC ink. A spatial phase modulator (SLM) imparts programmed phase delays to a coherent light source and is projected into the LC ink through an objective lens (60×, NA = 1.2). The 3D intensity light field (Figure 1a,b) is generated near the focal plane of the objective lens, where its maximum dimensions can reach up to approximately 150μm laterally and 100μm axially, while 5μm of feature size is achieved in the experiment. The LC ink formulation (Figure 1e and for details, see Experimental Section) contains C6BAPE (19.6 wt.%), RM105i (39.22 wt.%), A6OCB (39.22 wt.%) and TPO (1.96 wt.%) as inspired from [54]. Using a liquid crystalline diacrylate based on only two benzyl rings, C6BAPE, leads to lower transition temperatures [55] compared to systems containing the classical three benzyl rings [56]. A PVA coated LC cell is rubbed before filling the LC ink into the cell, as indicated by the red arrow in Figure 1c. The rubbing induces a global homogeneous alignment of the LC molecules in the cell, as shown in Figure 1c,e. Once the molecules are aligned in the cell, the nematic phase is retained during the fabrication at room temperature.

The fabrication of the infinity loop with a build volume of ≃ 160 μm 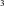, is limited by the shutter and takes a few milliseconds on our setup. The bright voxels of the holographic image are designed to exhibit intensity above photo‐curing threshold for the given exposure time (see Experimental Section), such that the photo‐polymerization initiates almost simultaneously throughout the volume. Because the object is printed as a single unit, it doesn't require sequential exposures for fabrication. This avoids the formation of director distortion typically seen in fabrication methods requiring sequential exposures (see Figures S1 and S2 and Video S1). The fixed director alignment within the polymerized network allows 3D LC structures to undergo reversible actuation in response to external stimuli, as schematically illustrated in Figure 1d. The structure contracts along the direction of the director and expands perpendicular to it, when its molecular alignment is distorted, for instance, upon heating. Moreover, rotating the projected 3D images in 3D enables to print structures with the same geometry but with different 3D director orientations, which results in objects with different actuation profiles.

**FIGURE 2:**
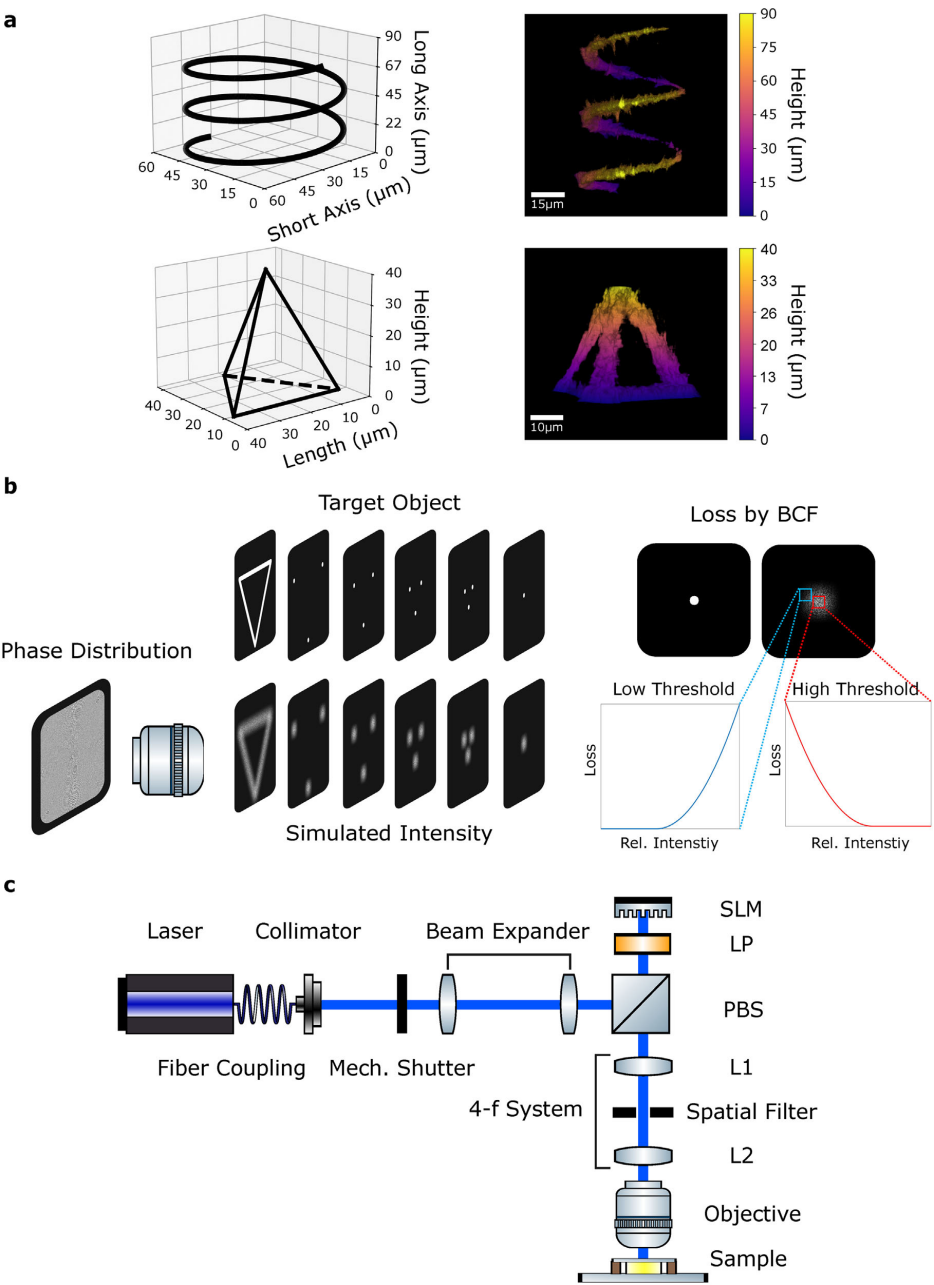
Generation of 3D light intensity pattern. (a) Reconstructed 3D light patterns in the shape of a spring (top) and a tetrahedron (bottom). The target images of each shape are presented on the left, and the corresponding 3D patterns are shown on the right. (b) Description of the NOVO‐CGH algorithm. A binary cost function (BCF) is employed to minimize unwanted photo‐polymerization outside the target regions, while it can promote simultaneous onset of photo‐polymerization throughout the target regions (c) Description of optical setup. SLM ‐ spatial light modulator, LP ‐ linear polarizer, PBS ‐ polarising beam splitter, L1/2 ‐ lens 1/2.

To calculate the required phase delays for generating the desired 3D images, we use an iterative phase‐retrieval CGH algorithm developed for non‐convex optimization problems (NOVO‐CGH) [53]. Figure 2b explains its optimization process. The NOVO‐CGH requires, first, a binary target object as an input. During each iteration, the light field is propagated from the SLM to the volume of interest and then compared to the target object, where any mismatch (loss) is quantified with a chosen cost function. The gradient of loss is calculated and used to determine the next guess of the phase distribution with the help of a Limited‐memory Broyden–Fletcher–Goldfarb–Shanno algorithm (L‐BFGS) [57]. The optimization is repeated until a wavefront can well reproduce the input target object. Throughout this study, a binary cost function (BCF) is used to calculate the loss. The BCF works with two thresholds that increases penalties if the simulated light intensities are not sufficiently low in dark regions or not sufficiently high in bright voxels, as shown in Figure 2b. We tune both threshold parameters to achieve shape fidelity of the target image and high intensity contrast between bright and dark regions.

The holographic microlithography is implemented with the optical setup shown in Figure 2c, (for details, see Experimental Section). A UV laser with tunable output power ranging from 0.6 to 100mW passes through a mechanical shutter operating at up to a speed of 25Hz, allowing to control the delievered energy for the photo‐polymerization with help of output power. The phase of laser is modulated by the spatial light modulator (SLM), then it is relayed through a 4‐f optical configuration, where a spatial filter is placed at the Fourier plane to remove the unmodulated components from SLM. Lastly, an objective lens (60×, 1.2NA) transforms the phase‐modulated components into 3D images, where it can illuminate maximally a volume of 152μm × 152μm × 113μm. By tuning the laser power and exposure time, it is possible to control the degree of curation in this volume.(Figure 2a) [44, 58, 59, 60, 61].

To confirm that the holograms generate 3D light intensity patterns that replicates well the target objects inside the medium (e.g. Figure 2a and Videos S2–S4), fluorescent images of a thin dye‐loaded layer are experimentally acquired along the optical axis. Then, the images are computationally stacked to directly visualize the 3D light pattern (see Experimental Section).

### 2.2 LC Structures With Independent 3D Director Alignement

**FIGURE 3:**
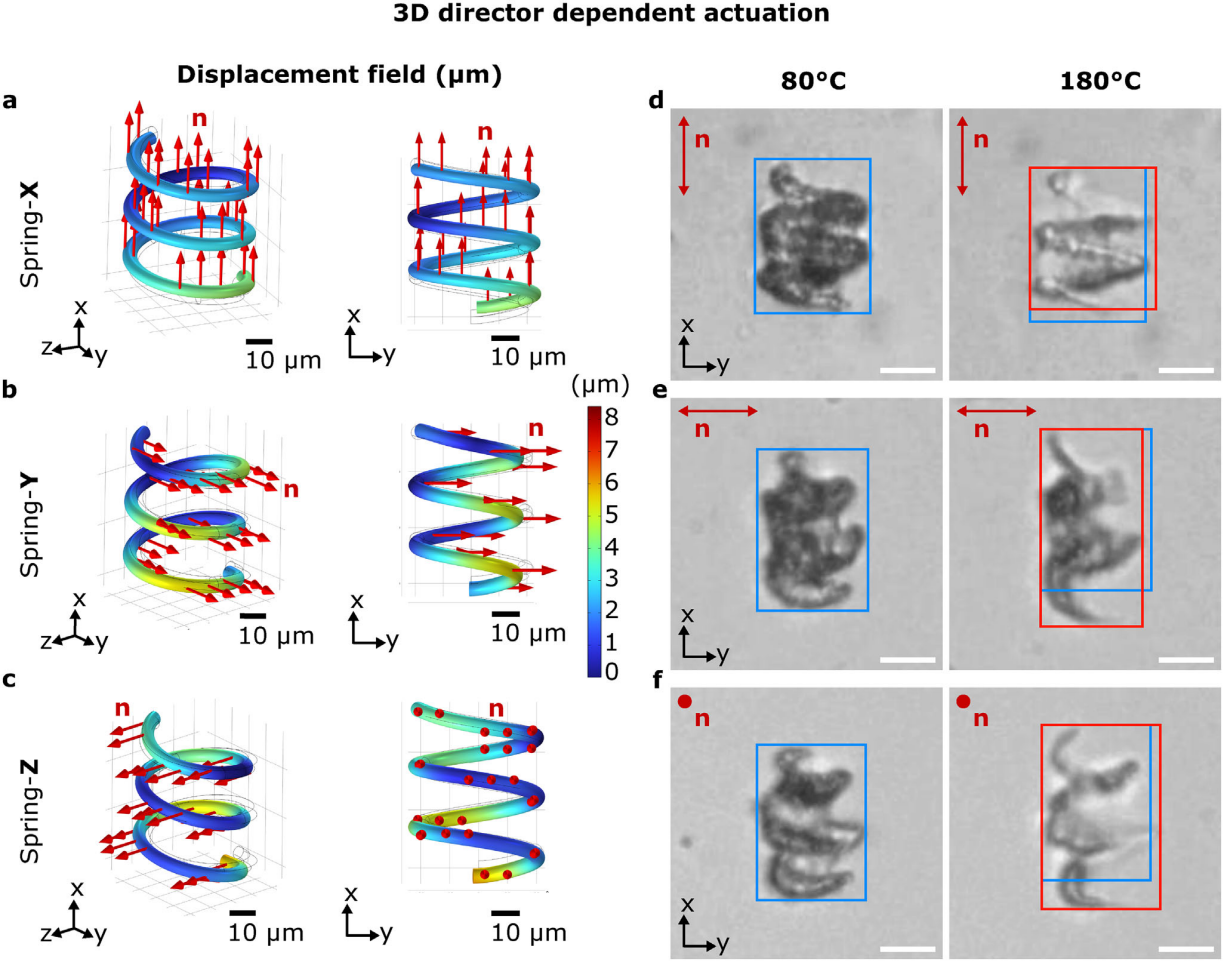
3D spring‐shaped LC structures whose actuation is determined by the 3D director alignment. (a–c) Simulation of the displacement field (in μm) in the 3D space and their 2D projection in the x‐y plane. The director orientation along x, y, and z axis is indicated by the red arrows for (a) Spring‐X, (b) Spring‐Y, and (c) Spring‐Z, respectively. On actuation, all the structures exhibit director‐dependent mechanical deformations, where the color scale bar shows the displacement fields (the length of the displacement vector) for all the elements of the printed geometry upon heating. (a) Spring‐X: with director along the spring's long axis, contracts along its body axis and expands perpendicular to it. (b) Spring‐Y: The director is aligned along the y‐axis, which is along the short axis of the helix. This spring contracts along its short in‐plane axis and expands orthogonal to it. (c) Spring‐Z: The director is aligned homeotropically along the z‐direction, and therefore, the structure expands along both the x‐ and y‐axes. (d–f): Observed thermal actuation of the LC Spring‐X, Spring‐Y and Spring‐Z, respectively. The blue and red bounding boxes indicate the outer dimensions of the structures at T = 80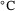 and 180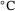, respectively, to facilitate the comparison of their mechanical deformations. In the right column, the boxes are aligned at the top left corner to show the extent of the deformation. The scale bars in (a–c) are 10μm and (d–f) are 20μm.

To show that the director can be fixed in the desired orientation within the printed structure, we prepared an LC cell with homogeneous planar alignment (see Experimental Section) and printed letters by a planar projection (Figure S3). As discussed in the Supporting Information, the molecular order is maintained after polymerization, and rotating the hologram image in plane results in a relative change in the director alignment with regards to the image orientation. Whole 3D structures can be printed in a single exposure using our setup (see Video S5), and since the printing geometry is controlled directly by the phase modulated light beam, the director orientation (set by the LC cell) can be decoupled from the axes of the target structure. Figure 3 shows three types of LC springs, all printed in the same orientation, but with different director alignments: LC Spring‐X and LC Spring‐Y are printed in homogeneous cells with the director aligned along the x and y directions, respectively, corresponding to the long and short axis of the helix. LC Spring‐Z is printed in a homeotropic cell, where the director points along the z‐axis (out of plane). After polymerization, the director dictates the actuation profile, together with mechanical boundary conditions such as rigid bonds to the substrate. To facilitate shape deformation during thermal cycling, the structures are purposely printed with only a few localized anchoring points on the glass substrate, allowing the rest of the structure to deform freely while remaining within the field of view.

The actuation behavior of these LC springs differs due to the director alignment, as demonstrated by our simulations (see Experimental Section). Figure 3 presents a comparison between the simulation results and experimental observations. In the simulations, the displacement field is calculated for each element of the LC structure, where the anchor points are fixed. The first column in Figure 3 shows 3D visualizations of the simulations, while the second column shows the corresponding 2D in‐plane projections. As expected, the LC springs exhibit contraction along the director and expansion perpendicular to it. Spring‐X contracts by ∼5% along the director and expands ∼6% perpendicular to it. Spring‐Y shows ∼4% contraction along the director and ∼17% expansion perpendicular to it. Spring‐Z, with the director oriented out‐of‐plane, expands by ∼5% and ∼15% in both in‐plane directions. This leads to distinct deformation patterns between springs, as highlighted by the blue and red rectangular bounding boxes corresponding to temperatures of 80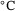 and 180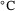, respectively. The data shows that the mechanical deformation of the structures depends on the director alignment relative to the body axis. This validates our fabrication scheme, where the global director alignment of a liquid crystal precursor is fixed independently in 3D inside the printed object.

**FIGURE 4:**
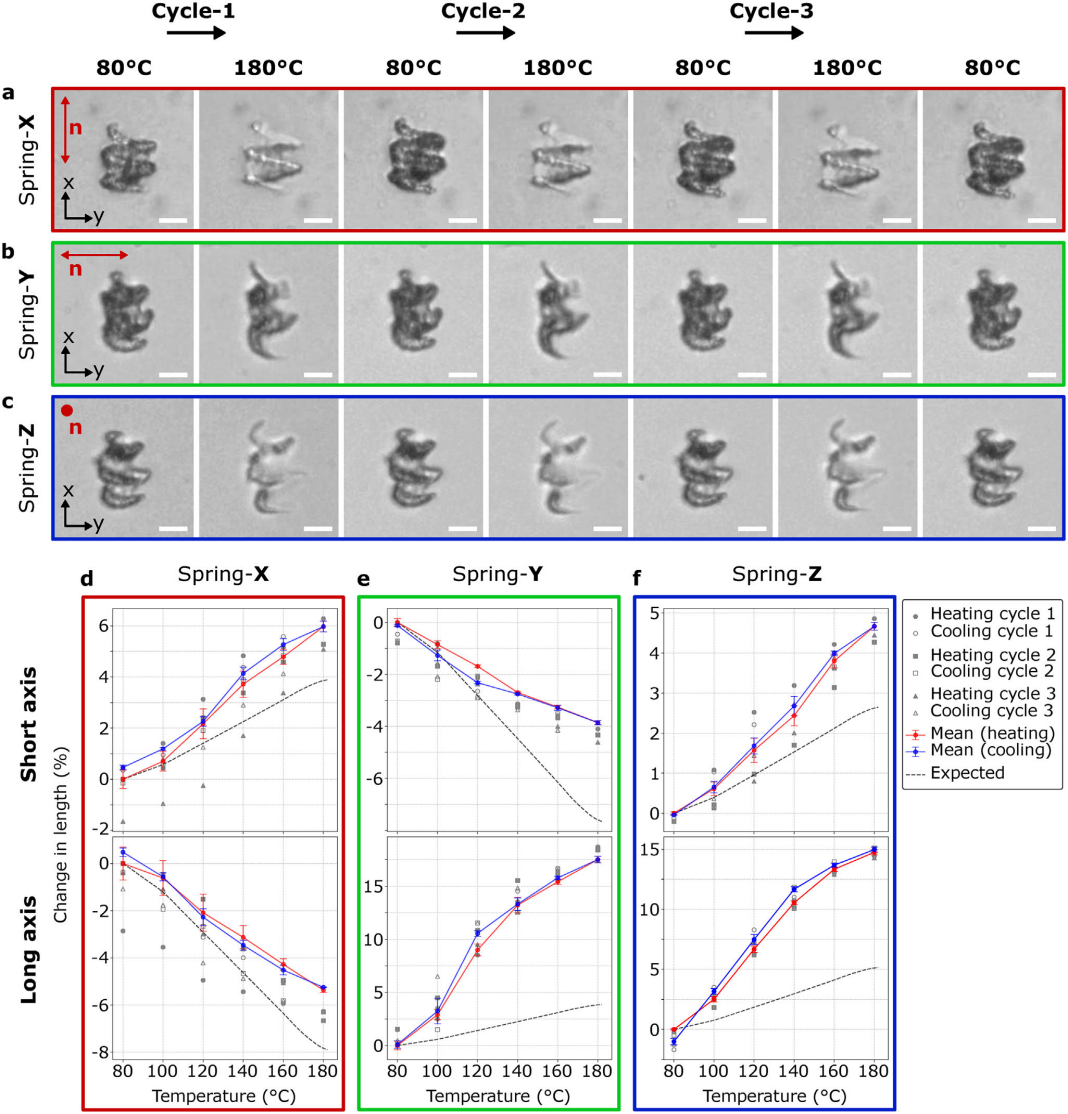
Reversible and repeatable actuation cycles of printed LC springs. (a–c) Observed actuation during three heating cycles for Spring‐X, Spring‐Y, and Spring‐Z, respectively, between $T=80^{\circ }C$ and $T=180^{\circ }C$. The director alignments are indicated with red markers in each of the images. (d–f) Plots for the measured deformation and simulation results for LC Spring‐X, Spring‐Y, and Spring‐Z, respectively. The mean percentage change in length for each heating and cooling actuation cycle is plotted with error bars representing the standard deviation, while the individual data points are shown as grey scatter plots. The simulation predictions are plotted by the dashed lines. Scale bars are 20 μm.

The structures return to their initial shapes upon cooling, as shown in Figure 4a–c (also Videos S6–S8 respectively). Repeated heating and cooling cycles show that the deformations are reversible (Figure 4d–f). The director‐dependent contraction or expansion agrees with the predictions. Similarly, this also holds true for both LC Spring‐Y and Spring‐Z (Figure 4e,f).

Experimentally observed actuation modes are in close agreement with finite‐element simulations that explicitly assume a uniform director field. Because the mechanical response of liquid crystalline elastomers is highly sensitive to local director orientation, this agreement provides additional indirect confirmation that the director remains well aligned throughout the structure, even in regions of high curvature.

### 2.3 3D Open Infinity LC‐Loops

**FIGURE 5:**
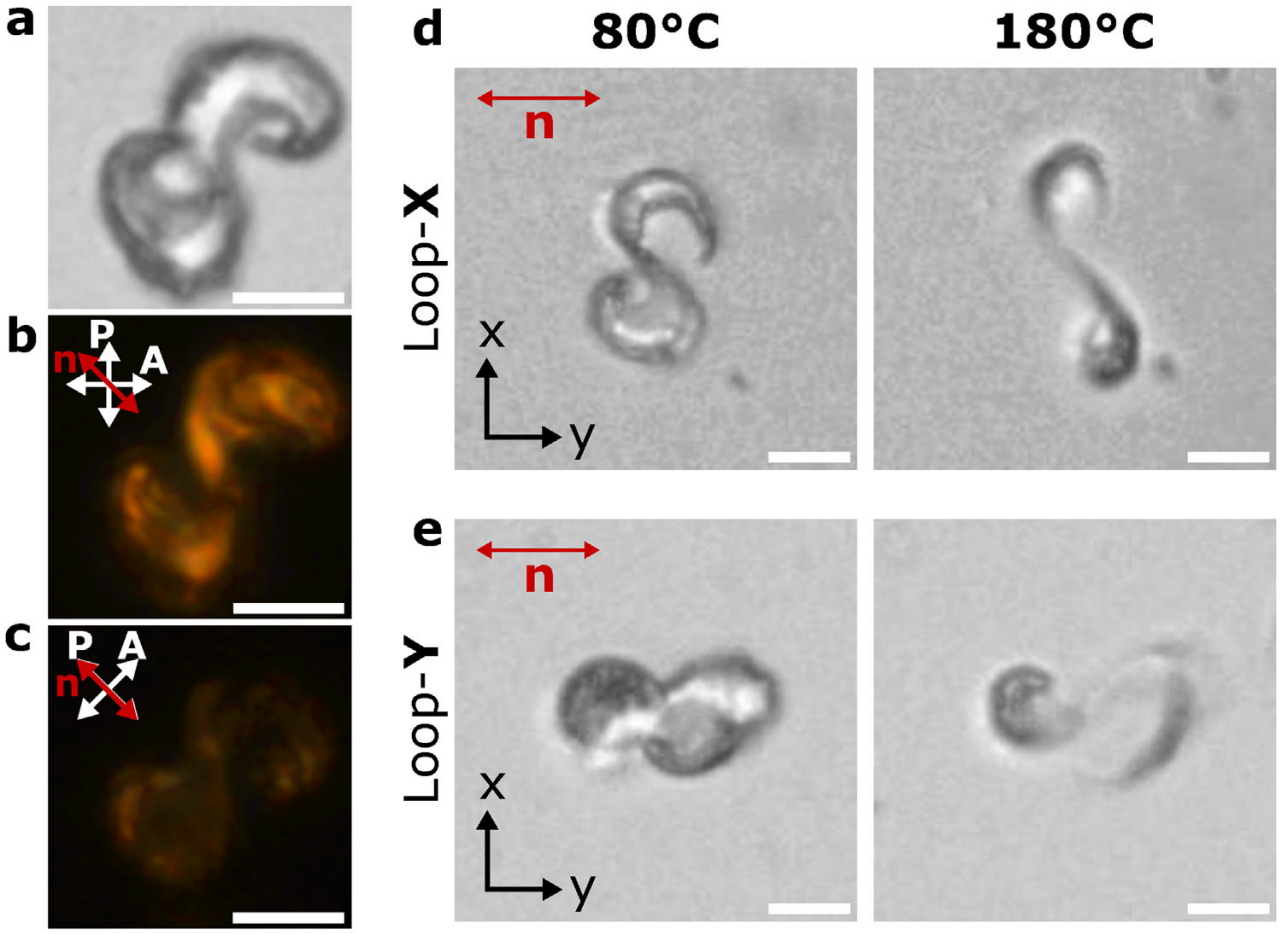
Director alignment and reversible actuation of printed 3D infinity LC‐loop. (a) Bright field image of the printed 3D infinity LC‐loop. (b,c) Polarized optical microscopy (POM) images are in agreement with a director alignment along the short axis, observed as dark and bright images under crossed polarizers. (d) Thermal actuation of Loop‐X, printed with its long axis along the x‐axis, contracts along the director (short axis) and expands along its long axis. (e) Thermal actuation of Loop‐Y, with its long axis aligned with the y‐axis (same as the director), contracts along its long axis, resulting in a different deformation compared to Loop‐X. Scale bars: 20μm.

We now investigate a connected 3D structure that is challenging to fabricate with other methods – the Möbius strip or infinity loop (Figure 5a). The loops are polymerized with the same light intensity patterns as shown in Figure 1, with a uniform global director alignment verified by polarization optical microscopy (POM) (Figure 5b,c). The loops were printed, anchored to the glass substrate, with an open end purposely designed for improved anchoring.

We examined the actuation behavior of two open LC loops—Loop‐X and Loop‐Y—printed with their long axes aligned along the x‐ and y‐directions, respectively, while the director remained fixed along y. As a result, both actuators contract along the director, which is orthogonal to the structural axis in each case, leading to distinct deformation behaviors (Figure 5d,e). This actuation response is further supported by simulations (Supporting Information, Figure S5 and Videos S9 and S10).

### 2.4 4D LC‐Flower

**FIGURE 6:**
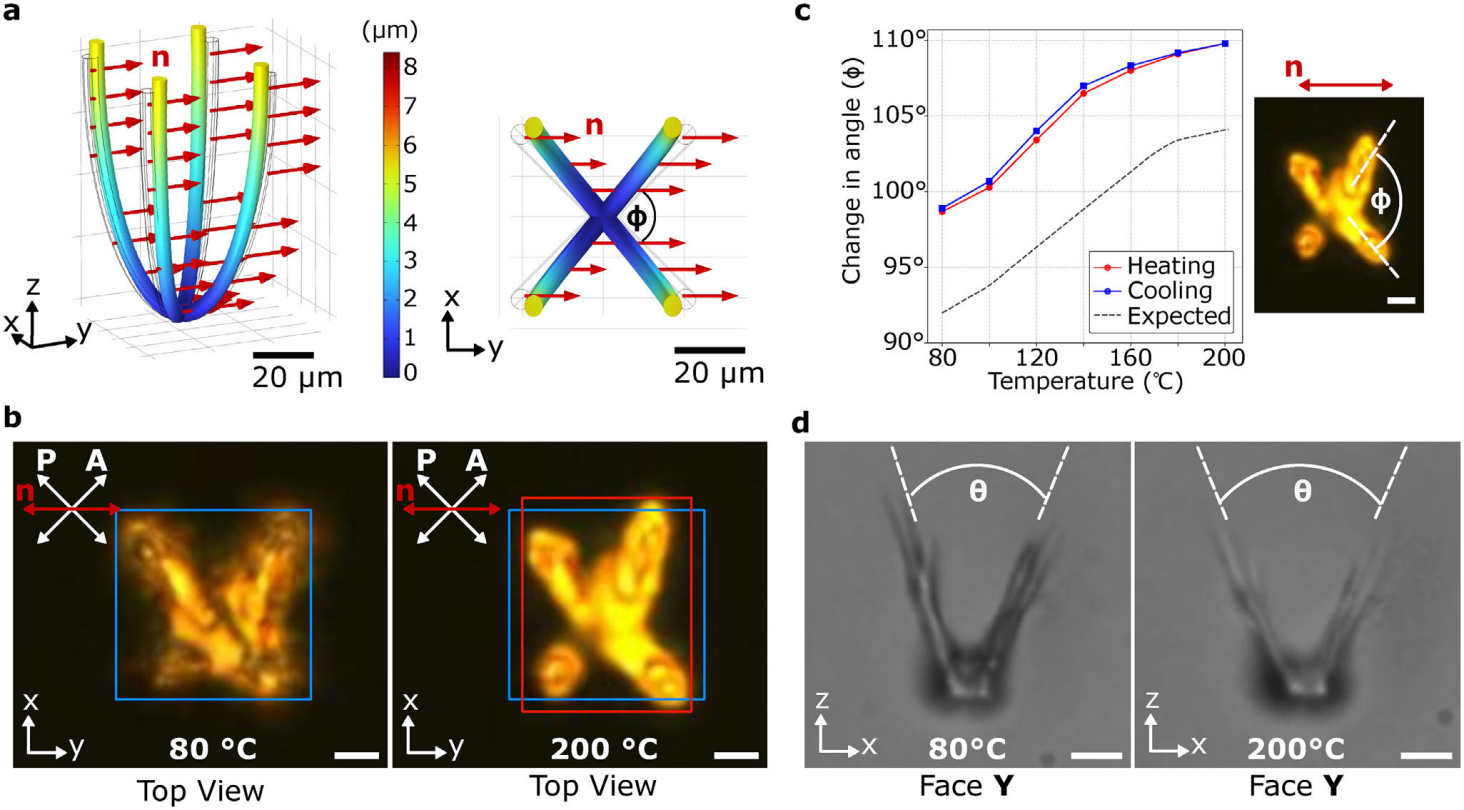
3D LC‐flower with four petals. (a) Simulation of the actuating LC‐flower structures in 3D and their 2D projection in the x‐y plane. The LC flower is printed with an in‐plane rotation, with the director aligned along the y‐axis, at a 45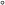 angle to the plane of the opposing petal pairs. The thermal actuation is measured under a POM at T = 80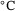 and 200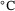. (b) Observed thermal actuation (top view) as the flower contracts along the director and expand perpendicular to it. The boxes are used to measure the outer dimensions of the structures at 80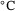 and 200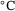, respectively. The blue box represents the original size of structures, while the red box in the right figure shows the deformed size. (c) The simulation and measurement of the angle φ are shown, illustrating how deformation changes the angle between the diagonal planes containing the pairs of opposing petals. (d) Side view in the x‐z plane shows the opening angle θ increasing upon actuation, as expected from the simulations. The scale bars are 20 μm.

A flower‐shaped LC structure was fabricated using the same method and anchored at the base, while its petals remain free to move. The petals' motion is based on their in‐plane orientation relative to the director alignment, as shown in Figure 6. The flower structure consists of two perpendicular pairs of opposing petals, as depicted in Figure 6a. The director alignment is at 45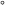 to the planes of both pairs, which can be considered as lying along the face diagonals of a cuboid. As predicted by the simulations in Figure 6a, the angle φ between the planes of the opposing petal pairs increases upon actuation. This change in angle can be interpreted as the deformation of the face diagonals of a cuboid that contracts along the director and expands perpendicular to it. In the experimental measurement, the blue and red bounding boxes denote the outer dimensions of the structures at 80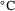 and 200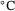, respectively. The observations from the top view are shown in Figure 6b and the plots for the change of the angle φ (measured from top view) are shown in Figure 6c. The side view offers another interesting insight as the opening angle θ appears to change upon actuation. Looking on to the x‐z plane (with director out of plane, along y axis), angle θ increases on actuation as observed in Figure 6d. The flower structure shows significant contraction and expansion in the orthogonal directions, indicating that the global director is fixed as desired in a complex 3D LC structure.

## 3 Conclusion

This work introduces volumetric holographic microlithography as a single‐exposure fabrication strategy for 3D LCE microstructures using inks with independently controlled global director orientation, opening new design possibilities beyond sequential photopolymerization methods. The method enables fabrication of complex geometries with a single‐shot exposure at room temperature, which is challenging to achieve with conventional fabrication techniques. The molecular alignment is preserved during the one step polymerization with a 3D light intensity pattern. We demonstrate our approach by forming various examples of LC microstructures with a feature size of 5μm, approximately, that exhibit director‐dependent actuation behaviors. The observed actuations agree well with structural deformation simulations from finite element modeling.

Holographic microlithography employs NOVO‐CGH algorithm to generate desired 3D light patterns, which then induce the photo‐polymerization of the LC. Without the need for any supporting materials the holographic projection setup can produce free‐standing structures within several milliseconds. Since the target object is polymerized as a single unit, the resulting structure does not show any interfaces within the object, which can disturb the director alignment. By rotating the projected 3D images, it is possible to create structures with the same shape but with different director orientation. We have shown that this results in distinct actuation profiles and the results match with numerical predictions.

On the other hand, the use of holograms also imposes limitations, as not all geometries can be realized with a single hologram. Constructing 3D structures from finite‐sized 2D phase arrays places constraints on the feature sizes, fidelity and complexity that may be realized. These constraints also result in some off‐target exposures at the margin of targeted regions. The choice of objective lens can influence the resolution of the printed structure and its overall dimensions, which restricts to realize a large object with fine details.

The throughput can be increased by combining our approach with a microfluidic flow to replenish the ink [62, 63]. Similarly, the holographic fabrication can print multiple objects in parallel by using the SLM as microlens arrays [45, 64, 65]. As it relies on single photon excitation for photo‐polymerization, our method is compatible with a wide range of ink formulations, envisioning the fabrication of structures responsive to various types of stimuli other than heat [24, 66, 67, 68]. More complex actuation profiles can be realized by spatially patterning the director field to create arbitrary 3D alignment within the structures [7, 27, 38, 69]. These ideas can be extended to actuators that consist of multiple sub‐sections for an enhanced actuation response. Our work shows that holographic microfabrication is a promising technique that can rapidly produce complex 3D liquid crystalline microactuators preserving molecular order.

## 4 Experimental Section

### 4.1 Optical Setup

The optical setup is shown in Figure 2c. A continuous‐wave 405nm diode laser (Oxxius LBX405–100) with a laser power of up to 100 mW was coupled to an optical fiber. The light passes a collimating lens, and the resultant Gaussian output beam has a diameter of 5mm and was passed through a mechanical shutter (Thorlabs SHB05T), capable of operating at a maximum speed of 25 Hz. The radiating power and mechanical shutter were used to control curing degree. A beam was expanded to the 8mm in diameter to match the screen size of the spatial light modulator (SLM). The laser beam was deflected by a polarizing beam splitter (PBS) and passes through a linear polarizer (LP) oriented at 45 degree, aligned with the active polarization axis of the SLM. The reflective phase‐only SLM (Pluto‐2‐NIR‐011) was installed to modulate the wavefront of laser. This high retardation SLM consists of 1080 × 1920 pixels, with a pixel pitch of 8 μm. Phase delays of larger than 2π at 405nm can be achieved with 8 bit resolution. After phase‐modulation by the SLM, the laser light passes back through the LP and PBS and was imaged by a 4f optical relay onto the back aperture of the objective lens. Since the reflected light from the SLM contains both modulated light and unmodulated light, a spatial filter was installed in the Fourier plane of the 4f system to block most of the unmodulated light. Lastly, an objective lens (Olympus UPlanSApo 60×/1.20W) transforms the modulated light into a 3D light pattern at its focal plane.

### 4.2 Binary Cost Function

We employ the NOVO‐CGH algorithm to compute phase arrays to generate 3D images. The NOVO‐CGH [53] is a phase retrieval algorithm optimizing 2D phase patterns that were transferred to the SLM. This phase pattern modulates the light to diffract so that it generates a 3D light pattern at a target distance. With a given phase information, the NOVO‐CGH simulates optical propagation through the volume of interest to determine the optimized phase distribution of the coherent light source. The optimization globally compares the simulated 3D image to the target image with a chosen cost function (loss function). The phase pattern was then updated based on the gradient of loss with Limited‐memory Broyden–Fletcher–Goldfarb–Shanno algorithm (L‐BFGS), and the process was repeated.

A loss was described as:

(1) $$L\left\{\phi \left(x,y\right)\right\}=\left\|{\left|F\left(A\left(x,y\right)\cdot e^{i\phi (x,y)}\right)\right|}^{2}-I_{\text{target}}\left(x,y,z\right)\right\|$$

where ϕ(x,y) was the phase pattern to be optimized, A(x,y) was the amplitude of the light at the phase modulator. F was a wave propagation operator, where the propagated amplitude was squared to compare with the desired 3D intensity pattern $I_{\text{target}}$. The ∥·∥ represents an arbitrary norm, evaluating the intensity mismatch between target object and simulated intensity. The NOVO‐CGH can accept user‐defined cost functions if they have well‐defined derivatives.

Because of the nonlinear threshold‐based photo‐polymerization, we chose binary cost function (BCF) which was based on L2‐norm. For regions should not be illuminated, $I_{\text{target}}\left(x,y,z\right)=0$, the binary cost function (BCF) was chosen to penalize any intensity above a threshold value (L):

(2) $$L\left\{\phi \left(x,y\right)\right\}=\left\{\begin{array}{cc} 0 & \cdots \;I_{\text{sim}}\left(x,y\right)\leq L\\ \sum {\left|I_{\text{sim}}\left(x,y,z\right)-L\right|}^{2} & \cdots \;I_{\text{sim}}\left(x,y\right)>L \end{array}\right.$$

where $I_{\text{sim}}\left(x,y,z\right)\;\text{is used for the}\;{\left|F\left(A\left(x,y\right)\cdot e^{i\phi (x,y)}\right)\right|}^{2}$. Similarly, if $I_{\text{target}}\left(x,y,z\right)=1$, the cost function returns penalties in case the intensity does not yet reach the intended threshold (H):

(3) $$L\left\{\phi \left(x,y\right)\right\}=\left\{\begin{array}{cc} \sum {\left|I_{\text{sim}}\left(x,y,z\right)-H\right|}^{2} & \cdots \;I_{\text{sim}}\left(x,y\right)<H\\ 0 & \cdots \;I_{\text{sim}}\left(x,y\right)\geq H \end{array}\right.$$

### 4.3 Reconstruction of Hologram Intensity Distributions

Fluorescent dye samples (Rhodamine B and Calcein dissolved in water) were employed to evaluate light intensity patterns of the 3D hologram images. A chamber filled with fluorescent dye solution (approximately 2μm in height) was placed at the focal plane of the objective lens, and the emitted fluorescence was imaged by a CMOS camera (CS2100M, Thorlabs). The produced 3D images were measured by two approaches: 1. A defocus algorithm was applied to the phase array to generate a virtual z‐stack of the 3D hologram. This algorithm effectively displaces the entire hologram along the optical (z) axis to scan different layers without moving dye sample mechanically. 2. A second objective lens was installed on the opposite side of the original objective. The second objective moves together with the dye sample to sequentially scan each layer of 3D image (see Videos S2–S4, and S11). The imaged z‐stacks were imported into ImageJ and rendered to create 3D reconstructions of the holograms.

### 4.4 Chemicals and Ink Preparation

The mesogens 4‐(6‐(Acryloyloxy)hexyloxy)phenyl 4‐(6‐(acryloyloxy)hexyloxy)benzoate (C6BAPE), 4‐Methoxybenzoic acid 4‐(6‐acryloyloxyhexyloxy)phenyl ester (RM105i), 6‐(4‐Cyanobiphenyl‐4'‐yloxy)hexyl acrylate (A6OCB) were bought from Synthon Chemicals GmbH & Co. KG. The photo‐initiator phenyl‐bis(2,4,6‐trimethylbenzoyl)phosphine oxide (TPO) was bought from Sigma–Aldrich. All the chemicals were used as received.

C6BAPE (19.6 wt.%), RM105i (39.22 wt.%), A6OCB (39.22 wt.%) and TPO (1.96 wt.%) were measured, then dissolved in 1 mL of dichloromethane (DCM) (Sigma–Aldrich) in a glass vial. The mixture was then heated and kept at 80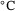 with continuous magnetic stirring for about 2 h. After all the DCM was evaporated, the mixture obtained has a nematic phase after naturally cooling down to room temperature.

### 4.5 LC Cell Preparation

The glass slides were cut into small pieces (size 4 cm × 2 cm) and cleaned sequentially by sonication in water, acetone, and isopropanol for 20 min each. After cleaning, the glass slides were dried using nitrogen and then plasma‐cleaned for 10 min. Subsequently, the slides were spin‐coated with a 5 wt.% aqueous solution of polyvinyl alcohol (PVA, 9000 Mw, Sigma–Aldrich) at 4000 rpm for 60 s and heated to 90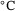 for about 10 min. After cooling to room temperature, the slides were uniformly rubbed along the desired director alignment direction using a velvet cloth. Finally, the LC cell was assembled using two different coated and rubbed glass substrates: one with a thickness of 1 mm and the other 150 μm, separated by 100 μm spacers.

### 4.6 Actuation Heating Cycles

For actuation measurements, a LTS‐420 Linkam heating stage was used to thermally actuate the LCs. The samples were heated at a rate of 30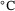/min from 80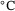 to either 180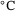 or 200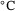. After reaching the target temperature, the heating was switched off, allowing the samples to cool naturally to 80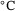. For imaging, the heating stage was mounted on a polarized optical microscope (POM) to only observe the polymerized structures within the LC cell, as the unpolymerized ink was in an isotropic phase at the elevated measurement temperature range.

### 4.7 Numerical Simulations

We developed a 3D model in *COMSOL Multiphysics* to simulate the thermal actuation of liquid crystal microstructures. The model construction was based on experimental data. We assume that the LC microstructure was in the nematic state in the reference configuration, with its directors uniformly aligned in a defined direction across the entire structure.

A uniform increase in temperature reduces the LC microstructures' order parameter and causing spontaneous deformations in both the parallel ($\lambda_{\left|\right|}$) and perpendicular ($\lambda_{⊥}$) directions relative to the liquid crystal's alignment (n). The LC microstructures displacement (u,v,w) due to the induced spontaneous stretches was computed by solving the mechanical equilibrium equation. which was expressed as:

(4) ∇·S=0

where S is the first Piola–Kirchhoff stress tensor, defined as the derivative of the elastic energy ($W_{\text{el}}$) with respect to the deformation gradient tensor (F):

(5) $$S=\frac{\partial W_{\text{el}}}{\partial F}$$

We assume that the elastic energy ($W_{\text{el}}$) of the nematic LCE is governed by the incompressible Neo–Hookean model.

(6) $$W_{\text{el}}=\frac{\mu }{2}\left(I_{1}\left(F_{\text{el}}^{T}F_{\text{el}}\right)-3\right)-p\left(\det\left(F_{\text{el}}\right)-1\right)$$

where μ is the shear modulus, and p is the Lagrange multiplier to ensure the incompressibility of the LCE. Also, $F_{\text{el}}$ is the elastic deformation gradient tensor, which was described by

(7) $$F_{\text{el}}=FF_{s}^{-1}$$

In Equation (7), $F_{s}$ is the spontaneous deformation gradient tensor with respect to the nematic state:

(8) $${\left(F_{s}\right)}_{ij}=\lambda_{\left|\right|}n_{i}n_{j}+\lambda_{⊥}\left(\delta_{ij}-n_{i}n_{j}\right)$$

Here, $\delta_{ij}$ represents the Kronecker delta, where $\delta_{ij}=1$ if i=j, and $\delta_{ij}=0$ if i≠j. The value of $\lambda_{\left|\right|}$ was determined experimentally for specific temperatures and interpolated for intermediate values (Figure S4). Additionally, $\lambda_{⊥}$ was calculated under the assumption of LCE incompressibility, satisfying the relation:

(9) $$\lambda_{\left|\right|}\lambda_{⊥}^{2}=1$$

Furthermore, fixed boundary conditions were applied to certain regions of each structure, based on experimental observations.

The governing Equation (4) were implemented in *COMSOL Multiphysics* to obtain the unknown displacements (u,v,w). Time‐stepping was handled using an implicit adaptive step‐size backward differentiation formula (BDF) solver. To address the non‐linear algebraic system arising from finite element discretization at each time step, a quasi–Newton algorithm was employed iteratively. The MUMPS direct solver was utilized for solving the linearized system during each iteration. Additionally, a mesh convergence study was conducted to ensure that the simulation results were independent of the mesh size.

For the simulations conducted, the material parameters were either extracted from previous papers or obtained by fitting the simulations to experimental results. The Young's modulus (E) of the LCF was assumed to be 4 MPa at room temperature. To simplify the modeling, the temperature dependency of the Young's modulus was neglected.

## Conflicts of Interest

The authors declare no conflicts of interest.

## Supporting information


**Supporting File 1**: adma72280‐sup‐0001‐SuppMat.pdf.

**Supporting File 2**: /adma72280‐sup‐0002‐Video S1.avi.

**Supporting File 3**: adma72280‐sup‐0003‐Video S2.avi.

**Supporting File 4**: adma72280‐sup‐0004‐Video S3.avi.

**Supporting File 5**: adma72280‐sup‐0005‐Video S4.avi.

**Supporting File 6**: adma72280‐sup‐0006‐Video S5.avi.

**Supporting File 7**: adma72280‐sup‐0007‐Video S6.avi.

**Supporting File 8**: adma72280‐sup‐0008‐Video S7.avi.

**Supporting File 9**: adma72280‐sup‐0009‐Video S8.avi.

**Supporting File 10**: adma72280‐sup‐0010‐Video S9.avi.

**Supporting File 11**: adma72280‐sup‐0011‐Video S10.avi.

**Supporting File 12**: adma72280‐sup‐0012‐Video S11.avi.

## Data Availability

The data that support the findings of this study are available from the corresponding author upon reasonable request.
