# Supplementary material for: Holographic Whole‐Object Photopolymerization Preserving Director Alignment in Liquid Crystalline Actuators
Source: Adv Mater. 2026 Jan 29;38(13):e19970. doi: 10.1002/adma.202519970 (PMC12957877; doi:10.1002/adma.202519970)
Supplement: Supplementary file 1 — Supporting File 1: adma72280‐sup‐0001‐SuppMat.pdf. [file ADMA-38-e19970-s009.pdf]

Supplementary Information

Holographic Whole-Object Photopolymerization Preserving Director Alignment in Liquid Crystalline Actuators

Lovish Gulati*, Junhee Lee*, Reza Norouzikudiani, Jianfeng Li, Carlos Sánchez Somolinos, Antonio DeSimone, Kai Melde, Alexander Song, and Peer Fischer†

*These authors contributed equally to this work.
†Corresponding author: peer.fischer@mr.mpg.de

Table of content

S1 Interfaces in sequential exposures

S2 Sequential v/s single-shot exposure

S3 2D structures with independent uniform global alignment

S4 Film contraction measurements upon actuation

S5 Actuation: 3D open infinity LC-loops

S1 Interfaces in sequential exposures

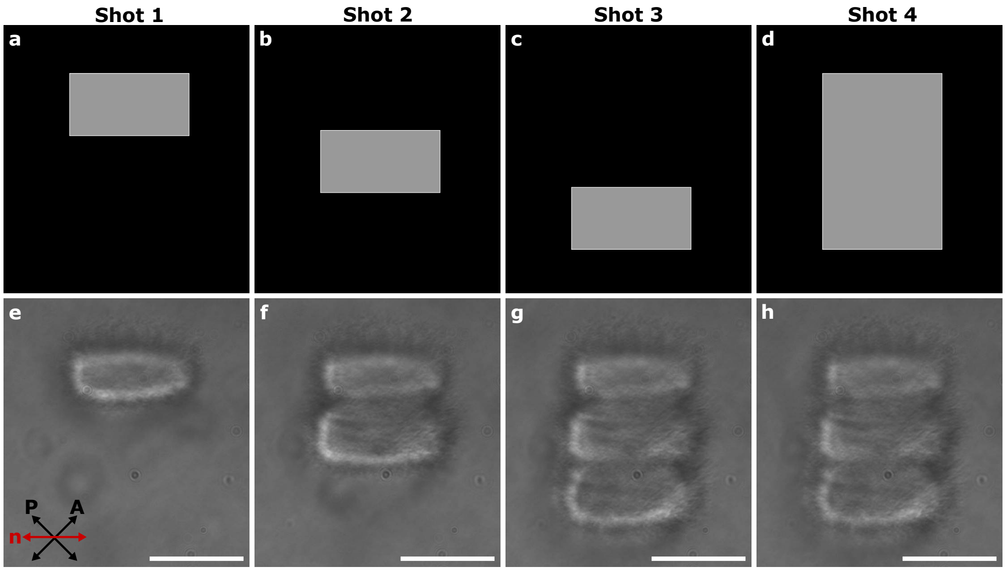

Figure S1: **Sequential printing to fabricate larger polymerized structures.** The target areas for the light exposure are indicated by the grey rectangles, where the exposed region is indicated by grey and the background regions in black receive no light. e-h) The resultant exposed regions showing the polymerized structure in bright field microscopy under polarized light. It is clearly seen that sequential stitching (g) results in misalignment at the interface, as observed in crossed polarizers. The interfaces between the sections are clearly visible. The final exposure in (d) is used to ensure that the sections are completely polymerized during exposures in (a-c). The scale bars are 20 μm.

S2 Sequential v/s single-shot exposure

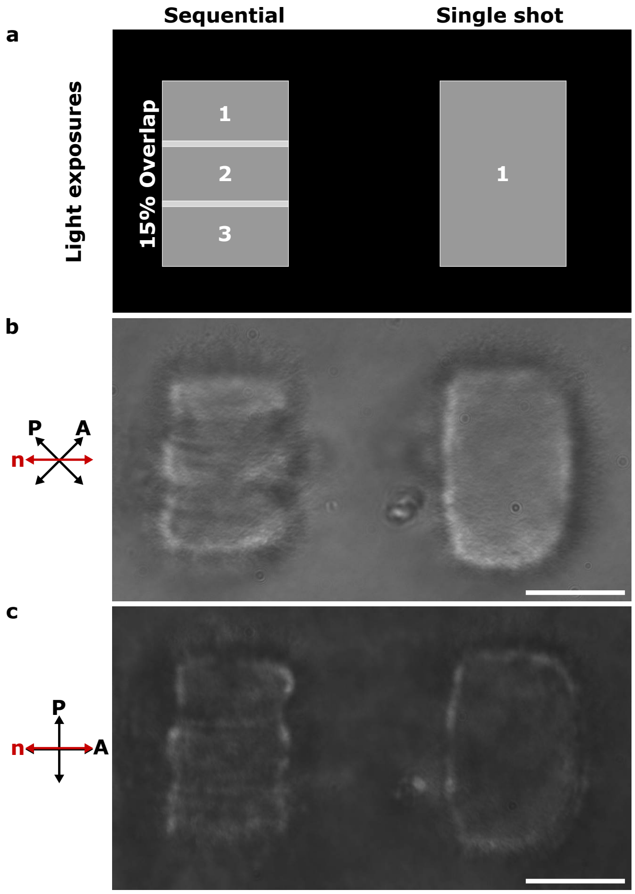

Figure S2: **Comparison of sequential vs. single-shot exposure when polymerizing LC structures.** a) The light exposure patterns used for each section, where the sequential approach polymerizes each section separately, and the single-shot approach uses just one exposure for all the sections collectively. The total exposed area in both cases is the same. However, there is a 15% overlap in the areas exposed in the sequential step so that the interfaces are completely polymerized. b-c) Polarization optical microscopy images of the printed structures. Single-shot exposure polymerizes all the sections uniformly without the formation of any interfaces, in contrast to the sequential approach where interfaces and director distortion at the interfaces can be easily observed when observed in bright field under crossed polarizers. The scale bars are 20 μm.

S3 2D structures with independent uniform global alignment

Holograms are calculated to project the letters A, B, C, and D into a cell containing a LC in the nematic phase with homogenous planar alignment. It is seen that the molecular alignment is preserved for different orientations of the hologram's projection is rotated. Figure S3 shows polarized optical microscopy (POM) images of the letters 'A', 'B', 'C', and 'D' with varying global director orientations after polymerizing the LC. Each row corresponds to in-plane director angles of 0°, 15°, 30°, and 45°, respectively. The director alignment (red arrow) and polarizer optic axes (black arrows) are schematically indicated for each row. The printed structures become anchored to the substrate during fabrication.

To verify that the director in the printed structures is fixed, we heated the sample to 80°C, ensuring that all unpolymerized LC ink surrounding the structures becomes isotropic. Only the polymerized regions remain visible in the LC cell when observed in the POM. The optical contrast is a function of the molecular orientation within the structures is preserved, confirming that the director alignment is successfully fixed during the illumination and polymerization step. In Figure S3a, the structures appear dark, indicating that the director alignment is along the axes of the polarizers. The contrast of the structures increases as the director is fixed at the increasing in-plane angles of 15°, 30°, and 45°. Slight misalignment or shape differences could result from imperfections in the LC preparation or deformation caused by heating to 80°C.

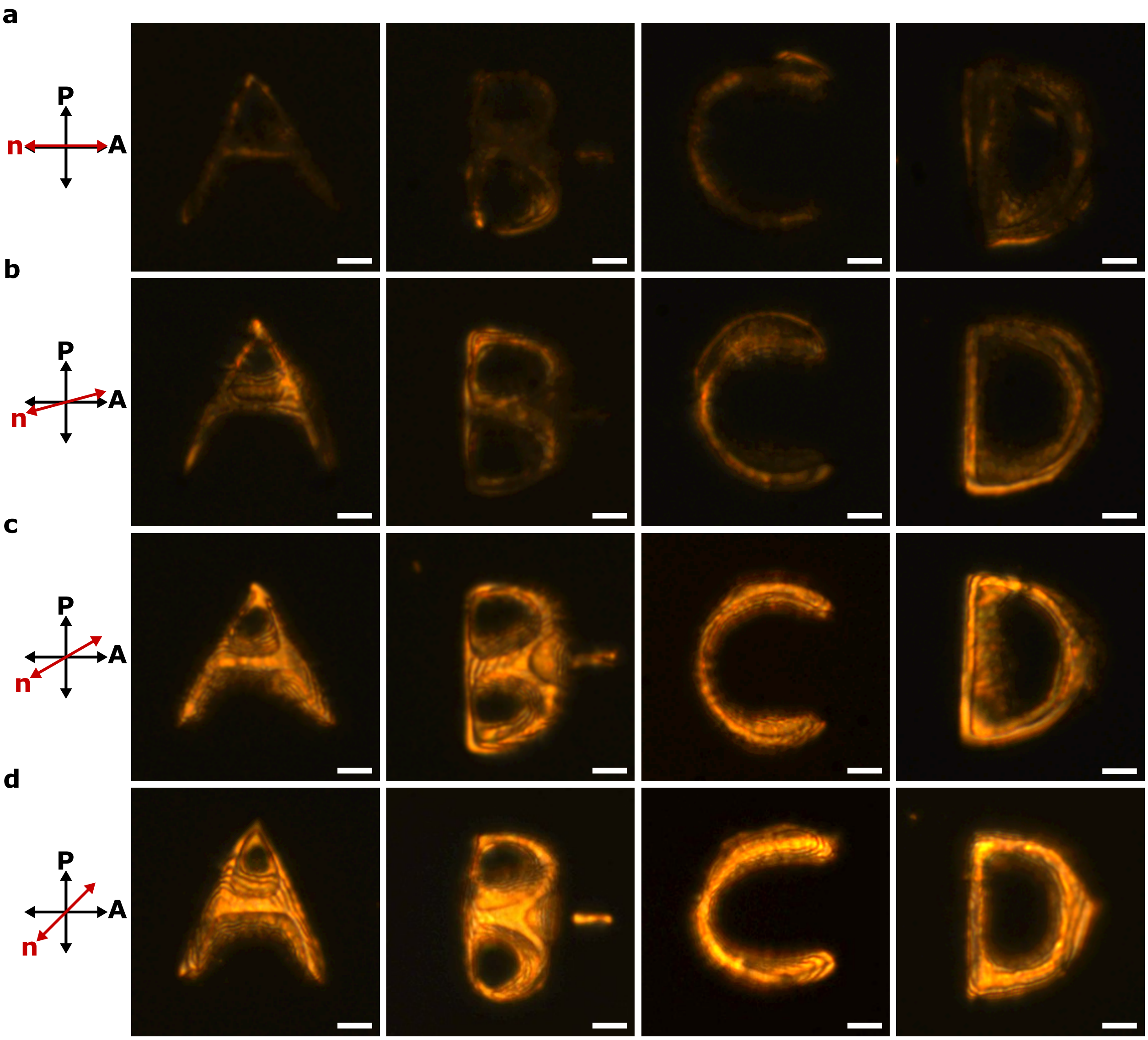

Figure S3: a-d) LC structures in the shapes of the letters A, B, C, and D, printed with different in-plane director alignments. The rows display structures where the alignment directions are, respectively, along $\varphi = 0^o$, 15°, 30°, and 45°. The polarizer and analyzer configurations are shown in black, while the director alignment is indicated by the red arrow. The scale bars are 20μm.

S4 Film contraction measurements upon actuation

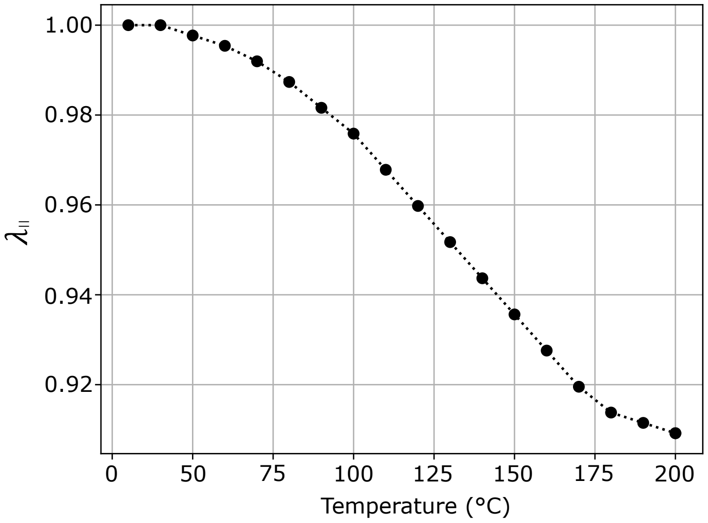

Figure S4: Measurement of the contraction of the LC film with uniform in-plane director alignment along its long axis under thermal actuation. The film contracts by about 9% along the director.

S5 Actuation: 3D open infinity LC-loops

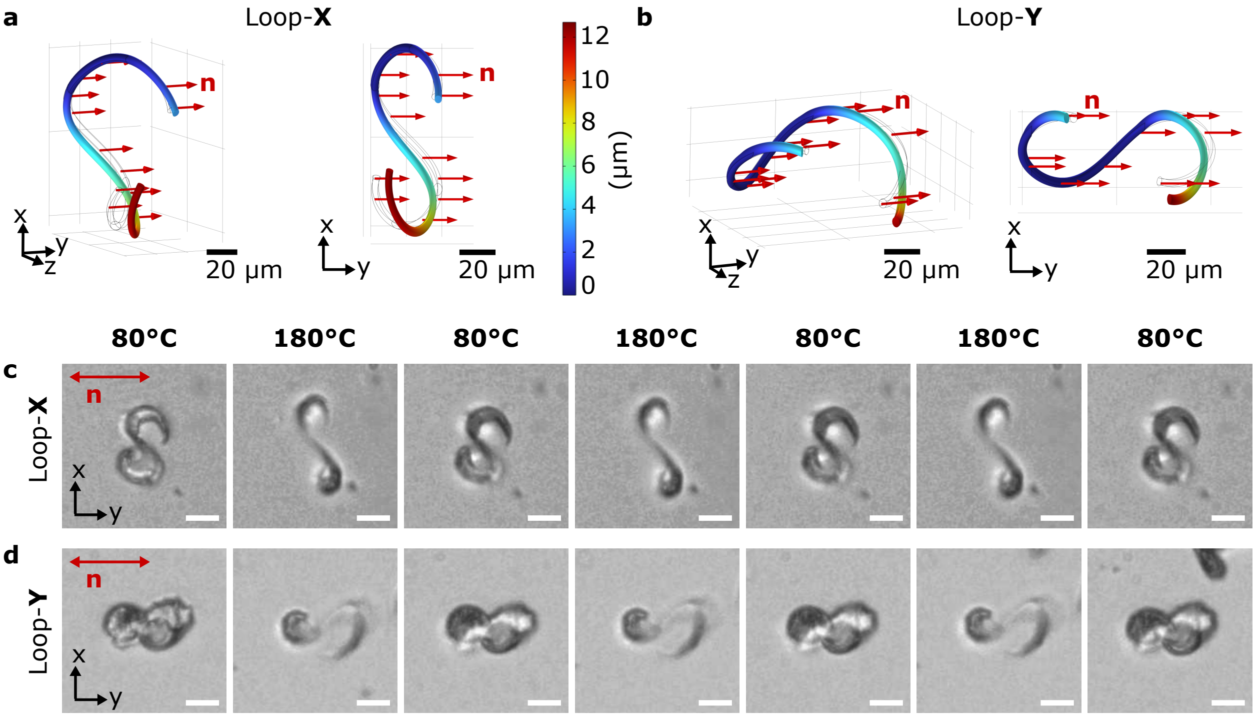

Figure S5: **Complex 4D LC-loops.** a-b) Simulation visualizations of the complex 4D LC-loops, shown in 3D and as 2D projections in the x-y plane. The director '**n**' is aligned in the y-direction in both cases and the shape orientation of the printed structure is directly controlled by the projected hologram image. c-d) Observed thermal actuation over three consecutive heating–cooling cycles between T = 80°C and 180°C for LC loop-X and loop-Y respectively. The actuation behavior depends on the director orientation relative to the body axis. The scale bars for both simulations and experimental data are 20μm.
